# Repetitive Bleomycin-Based Electrochemotherapy Improves Antitumor Effectiveness in 3D Tumor Models of Conjunctival Melanoma

**DOI:** 10.3390/jcm12031087

**Published:** 2023-01-30

**Authors:** Joana Heinzelmann, Sabine Hecht, Alexander Ruben Vogt, Udo Siebolts, Peter Kaatzsch, Arne Viestenz

**Affiliations:** 1 Department of Ophthalmology, University Hospital Halle-Wittenberg, 06120 Halle, Germany; 2 Institute of Pathology, University Hospital Halle-Wittenberg, 06112 Halle, Germany; 3 Institute of Pathology, University Hospital Köln, 50996 Köln, Germany

**Keywords:** conjunctival melanoma, electrochemotherapy, tumor spheroids, bleomycin, antitumor therapy, cytotoxicity

## Abstract

Background: Conjunctival melanoma (CM) is associated with a high rate of local recurrence and poor survival rate. Novel therapeutic options are needed to reduce recurrence rate. The objective of the study was to demonstrate the improved effectiveness of electrochemotherapy (ECT) on CM using repetitive application. Methods: Tumor spheroids of three CM cell lines (CRMM1, CRMM2, CM2005.1) were treated repetitively with ECT using the chemotherapeutic agent bleomycin on days 3, 5, and 7 of culture. Application of bleomycin alone and electroporation alone served as controls. The cytotoxic effect was analyzed on day 10 compared to untreated control using an independent t-test. The spheroid outgrowth rate was measured. Result: CM tumor spheroid size (median value: 78%, SD: 32%) and viability (median value: 11%, SD: 11%) were dramatically reduced after repetitive ECT treatment (*p*-value < 0.001). Decreased proliferation capacity (down to 8%) and an increase of apoptotic cells were observed. In most repetitive ECT-treated spheroids, no viable or proliferating cells were detected. Only 33–40% of repetitive ECT-treated spheroids exhibited single outgrowing cells with a delay of time up to 38 days. Conclusion: Repetitive ECT application effectively induces cytotoxic effects in CM spheroids by inducing apoptosis, inhibiting proliferation and decreasing the percentage of surviving tumor cells. Thus, repetitive ECT results in improved antitumor effectiveness in CM and could be an alternative therapy option.

## 1. Introduction

Conjunctival melanoma (CM) is a rare tumor arising from atypical melanocytes in the basal layer of the conjunctiva. As multiple studies have shown, it represents 2–5% of all ocular tumors and 5–7% of all ocular melanomas. The incidence is approximately 0.2–0.5 per million people in the Western world, with an increasing trend, likely due to increased UV exposure [1,2,3,4].

CM has similarities with cutaneous and other mucosal melanomas regarding genetic and pathophysiological features and differs from uveal melanoma. Similar to cutaneous melanoma, CM is characterized by mutations in the BRAF (up to 50%), NRAS (20%), and KIT (7%) genes and a UV light-sensitive signature (high burden of C→T mutation) [5,6,7]. NF1 mutations are described in 33% of CM [8]. Characteristic mutations for uveal melanoma in genes such as GNAQ, GNA11, and BAP1 have not been found in CM [6,9,10,11].

As CM is a rare cancer, tumor management is based on case series data and consensus opinion regarding surgical and medical management practices. The current gold standard treatment is surgical excision, often followed by various adjuvant therapies (such as cryotherapy, radiotherapy, or topical chemotherapy) due to the high recurrence rates [4]. Despite treatment, local recurrence develops in up to 66% of CM patients [12]. A total of 17–31% and 22–59% of patients will die due to the disease within 5 and 10 years, respectively [13]. Thus, novel therapy options are needed to improve the recurrence rate of CM.

By searching for alternative tumor therapy approaches with high effectiveness and high compatibility, we also focused on cancer types that share many genetic and pathophysiological features with CM, including cutaneous melanoma. Interestingly, electrochemotherapy (ECT) is one of the promising clinically verified therapy options for subcutaneous and cutaneous tumors and metastasis. ECT is a combination of chemotherapy and electroporation for local therapy of tumors. Electric pulses are used to reversibly permeabilize cell membranes to improve the intracellular uptake of poorly permeant or nonpermeant chemotherapeutic agents and to increase the local cytotoxic efficacy. The physical and chemical mechanisms underlying this approach have not been completely clarified. However, it has been shown that electric impulses induce the temporary opening of aqueous pores on the cell membrane of tumor cells, which allows the passage of genes and molecules [14,15,16]. Among several drugs tested in in vitro experiments and preclinical studies, bleomycin and cisplatin have shown the highest potential cytotoxic effect in combination with electroporation [15]. ECT is a local and nonthermal tumor therapy approach that is currently used in clinical practice to treat different tumors, such as soft tissue sarcomas and carcinomas, cutaneous melanoma, colorectal liver metastases, tumor nodules in the proximity of important structures such as vessels and nerves and tumors not amenable to excision or radiofrequency [17]. In addition, promising results have been achieved for application in deep-seated tumors of the liver, bone metastases, unresectable pancreas cancer, breast cancer, oral carcinomas, and periocular basal cell carcinomas [15,18,19,20,21,22]. Therefore, ECT is used as an alternative approach or in a palliative setting to improve the quality of life for patients. ECT can also be applied as neoadjuvant treatment in the form of cytoreductive therapy or as organ- or function-saving treatment [23,24,25,26]. In addition to the effectiveness, a low adverse event profile, tissue preservation, brief hospitalization, repeatability, and favorable cost–benefit ratio are further advantages of this therapeutic approach.

The impact of ECT in the management of CM is still not sufficiently clarified. However, the first in vitro studies have reported promising results [27,28]. These studies verified the cytotoxic effect on CM cells after a single application of ECT treatment using bleomycin. Recent studies on cutaneous and subcutaneous tumor lesions have shown a significantly improved effect of ECT after repeated application.

The objective of the study presented here was to demonstrate the increased effectiveness of electrochemotherapy (ECT) on CM using repetitive application. For this purpose, we used three-dimensional (3D) models of CM cell lines growing as tumor spheroids to assess the cancer related characteristics of CM of solid tumors in vitro. Such 3D models recapitulate the complexity and plasticity of the biological behavior of human cancers more similar to in vivo conditions than two-dimensional (2D) monolayer cultures, especially drug sensitivity and resistance, as shown for several cancer tissues [29,30,31]. In bulk tumors, the spatial structure of the cells, the presence of extracellular matrix proteins as well as several effects like limited oxygen diffusion and nutrient gradient can influence the effect of drug sensitivity of tumor cells [32,33]. In contrast to 3D models, in monolayer cell cultures, the effects of electroporation and the access to the chemotherapeutic agent is artificially equal for all cells, which failed the complexity of the in vivo environment. Therefore, tumor spheroids reflect the in vivo conditions of tumor behavior in a more comparable manner. Spheroids were created under non-adherent conditions and treated with ECT for three times. To measure the benefit of the repetitive ECT treatment, spheroid size and morphology, viability, proliferation, apoptosis as well as tumor outgrowth capacity were measured.

## 2. Materials and Methods

### 2.1. Culturing of CM Cell Lines

The CM cell lines CRMM1, CRMM2, and CM2005.1 were originally established from recurrence patients [34,35] and provided by the Liverpool Ocular Oncology Research Group. The authentication of the cell lines was performed using genotyping (Cellosaurus STR similarity search tool [36]) and negatively tested for mycoplasma infection using real time PCR [37]. Cells were cultured in complete medium containing F-12K medium with 10% FCS at 37 °C in a fully humidified atmosphere with 5% (*v*/*v*) CO_2_. Spheroid generation was performed as described in previous publications [28,38]. Briefly, spheroids were generated by seeding 5 × 10^3^ cells in round bottom 96-well ultralow attachment plates (Corning, Corning, NY, USA) containing 200 µL of complete medium.

### 2.2. Treatment of Spheroids

Three-day tumor spheroids were used as the starting point of treatment. Tumor spheroids were treated either with 200 µL complete medium containing 2.5 µg/mL bleomycin alone (chemotherapy), with high-voltage electrical pulse electroporation (750 V/8 pulses) in the absence of bleomycin (electroporation, EP) or with high-voltage electrical pulse electroporation in the presence of 2.5 µg/mL bleomycin (electrochemotherapy, ECT) using an approved voltage pulse generator (Cliniporator, IGEA S.p.A., Carpi, Italy). Details of the EP protocol are as follows: two parallel aluminum electrodes 4 mm apart, eight pulses, 100 µs pulse duration, 5 Hz repetition frequency, and 750 V/cm pulse strength. As a negative control, a further sample remained untreated (control). Four hours after treatment, the tumor spheroids were incubated in fresh complete medium.

**Figure 1:**
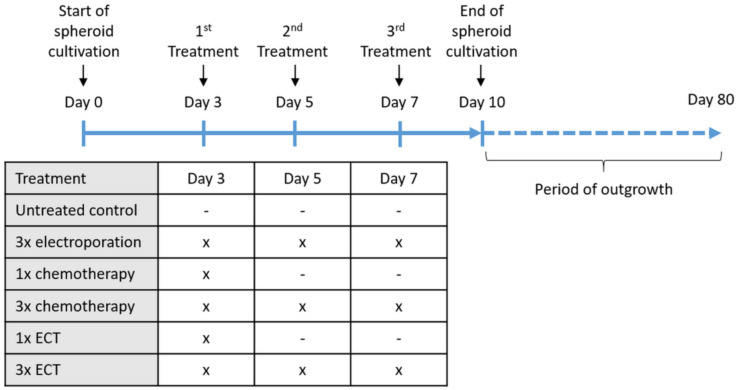
Graphical overview of treatment over time. Abbreviations: ECT = electrochemotherapy.

In this study, two different treatment options were used (Figure 1). Single treatment of chemotherapy and ECT was performed on day three of tumor spheroid culture (1× chemotherapy, 1× ECT). Repetitive treatment included three time points of treatment on days 3, 5, and 7 of tumor spheroid culture (3× chemotherapy, 3× EP, 3× ECT). Spheroids were harvested on day 10 of treatment for analysis. The analysis was performed with eight biological replicates per day at three to eight different time points.

### 2.3. Determination of Spheroid Growth

The growth of CM cell lines was analyzed on day 10 of spheroid cultivation using bright field microscopy (using an Axio Vert A1 microscope) by measuring the cross-sectional area of the spheroids as described in [28]. Therefore, the cross-sectional area of the spheroids was calculated using image-processing software ImageJ Fiji (Dresden, Germany) [39]. The relative treatment response was calculated by comparing the percentage of the median cross-sectional area of the samples to the median cross-sectional area of untreated control samples.

### 2.4. Determination of Spheroid Viability Using ATP Measurement

To calculate the viability of the tumor spheroids after 10 days of spheroid cultivation, we used a cell viability assay suitable for 3D cell cultures, namely, CellTiter-Glo 3D Cell Viability Assay (Promega Corporation, Fitchburg, WI, USA), according to the manufacturer’s instructions. Using the assay, spheroids were lysed, and viability was measured by quantification of ATP, which signals the presence of metabolically active cells. The relative treatment response was determined by comparison of the percentage of median viability of the treated samples compared to untreated controls.

### 2.5. Immunocytochemistry

On day 10, spheroids were carefully washed using PBS, fixed by incubating in 4% paraformaldehyde for 30 min and embedded in paraffin. Serial sections (4 µm) were prepared for immunocytochemical staining. Paraffin sections were incubated at 60°C for 2 h and then dehydrated with xylene and decreasing concentrations of ethanol, followed by distilled water. Antigen retrieval was performed using Tris/EDTA buffer (pH 9) for 30 min in a water bath at 95 °C. The endogenous peroxidase activity was blocked by using 3% H_2_O_2_ for 15 min. Sections were permeabilized and blocked with 2% BSA/10% FKS with 0.1% TBS/Tween 20 for 30 min at RT and incubated overnight with primary rabbit monoclonal antibody against cleaved poly(ADP-ribose) polymerase 1 (cParp-1) (1:1000, E51, Abcam, Cambridge, UK) or Ki67 (1:100, SP6, Thermo Fisher Scientific, Rockford, IL, USA) at 4 °C. The next day, sections were rinsed with TBST followed by assessment with an HRP/DAB detection system (Mouse/Rabbit PolyVue™ HRP/DAB Detection System, Zytomed Systems GmbH, Berlin, Germany). Sections were extensively rinsed with TBST, counterstained with Mayer’s hematoxylin solution (Merck, Darmstadt, Germany) for 2 min and mounted with Prolong Gold Antifade Mountant (Thermo Fisher Scientific, Rockford, IL, USA).

### 2.6. Outgrowth Capacity of Treated Spheroids

To test outgrowth capacity after 7 days of treatment, spheroids were carefully transferred to 24-well cell culture plates in a group of four spheroids/well (Figure 1). The medium was carefully changed every 2–3 days. The outgrowth capacity was monitored on a daily basis on microscopic images of cell culture plates using an Axio Vert A1 microscope (Carl Zeiss AG, Oberkochen, Germany). The vital and proliferating cells were obtained, multiplied and cryopreserved for further analyses. When proliferating cells had not been detected within 80 days of outgrowth time, cultivation was stopped, and tested spheroids were defined to have no outgrowth capacity.

### 2.7. Statistical Analysis

All data are expressed in terms of the median value and range. An independent t-test was used to verify statistically significant differences between different treatment subgroups. Therefore, Levene’s test was used to proof that the variance is equal across treatment groups. Using the two-tailed test, a *p*−Value of 0.05 to 0.01 was defined as statistically significant, a *p*−Value of 0.01 to 0.001 was defined as statistically very significant, and a *p*−Value < 0.001 was defined as statistically extremely significant. All analyses were performed using the program SPSS Statistics 25 for Windows (SPSS Inc., Chicago, IL, USA).

## 3. Results

### 3.1. Spheroid Growth Is Significantly Reduced in CM Cells after Repetitive ECT Treatment

Based on previous results showing that a single application of ECT (1× ECT) had a cytotoxic effect on CM, we investigated the possible improvement of this therapeutic approach using repetitive applications of ECT. CM cells (CRMM1, CRMM2, and CM2005.1) were cultured as tumor spheroids for a period of ten days to mimic in vivo 3D tumor characteristics. At different time points, spheroids were treated with ECT (Figure 1). Untreated spheroids and spheroids treated with EP or chemotherapy alone were tested in parallel as controls.

Representative microscopic images of spheroids from the CM cells during the treatment period demonstrated different response characteristics depending on the treatment (Supplementary Materials Figures S1–S3). Compared to the untreated condition, 3× EP or chemotherapy (1× chemotherapy, 3× chemotherapy) alone was associated with comparable morphology and spheroid size. Treatment using 1× ECT resulted in a reduced size of 3D tumor aggregates, lower compactness with more loosely associated cells and a high amount of cell debris in the surrounding area. This effect was significantly increased by repeating this procedure (3× ECT).

**Figure 2:**
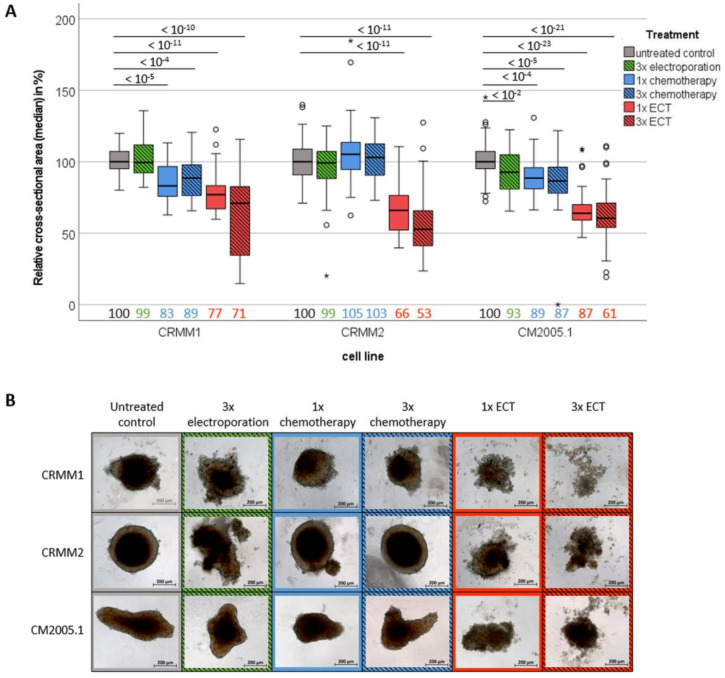
Comparison of spheroid size of different conjunctival cell lines on day 7 after different treatment options. (**A**) Boxplot of the relative cross-sectional area after different treatment options compared to untreated controls. Median values are listed on the bottom side of the boxplot. Statistical differences are stated above using *p*−Values (*); (**B**) Representative live images of spheroids after different treatment options. Scale bar = 200 µm. Abbreviations: ECT= electrochemotherapy.

To quantify the cytotoxic effect of 3× ECT compared to 1× ECT treatment, we first calculated the relative spheroid growth of different CM cells (CRMM1, CRMM2, CM2005.1) by measuring the cross-sectional area of spheroids after 7 days of treatment (day 10 of cultivation). In all three cell types, compared to the untreated condition and the single or triple treatment using EP or chemotherapy alone, ECT showed a strong significant reduction of spheroid size (Figure 2A). In CRMM1 cells, the median spheroid size after 1x ECT treatment was decreased to 77% (*p*−Value < 10^−11^) that of the untreated controls and 71% (*p*−Value < 10^−10^) that after three courses of ECT treatment (3× ECT). In CRMM2 cells, the median spheroid size was reduced to 66% (*p*−Value < 10^−11^) and 53% (*p*−Value < 10^−11^), respectively. For CM2005.1 cells, the median spheroid size amounted to 64% (*p*−Value < 10^−23^) and 61% (*p*−Value < 10^−21^), respectively. Compared to this, separate treatment with either 3× EP, 1× chemotherapy, or 3× chemotherapy resulted in only a weak reduction of spheroid growth in CM cells. The median values of all cell lines were 97%, 91%, and 90%, respectively. In addition, notable morphological differences were determined in ECT-treated spheroids after 7 days of treatment. The spheroids were flat and had frayed edges and many cell residues in the surrounding region, whereas spheroids after any other treatment maintained mostly a compact shape with smooth edges, as seen in Figure 2B.

### 3.2. Spheroid Viability Is Significantly Decreased in CM Cell Lines after Repeated ECT Treatment

**Figure 3:**
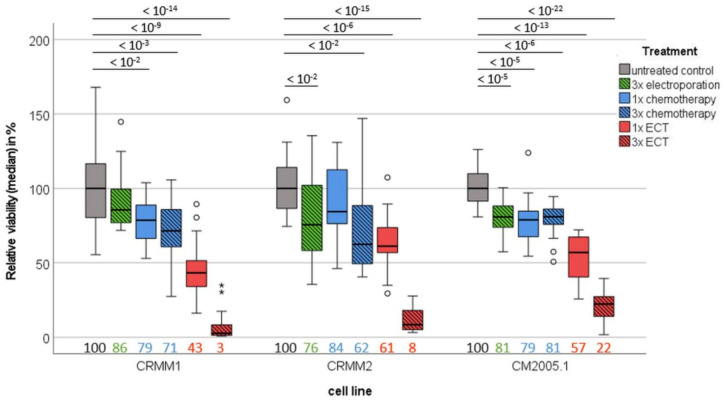
Comparison of the viability of different conjunctival cell lines on day 7 after different treatment options. Boxplot of the relative viability of different treatment options compared to untreated controls. Median values are listed on the bottom side of the boxplot. Statistical differences are stated above using *p*−values. Abbreviations: ECT = electrochemotherapy.

To prove the cytotoxic impact of 3× ECT, ATP concentration as an indirect measure of tumor spheroid viability was detected after the different treatment options compared to the untreated control. Therefore, 3× EP, 1× chemotherapy and 3× chemotherapy reduced the viability to 89% (SD: 20%, *p*−Value < 0.001), 97% (SD: 31%, *p*−Value > 0.05), and 99% (SD: 46%, *p*−Value > 0.05) of the initial median viability, respectively. Further, 1× ECT reduced the viability to 59% (SD: 30%, *p*−Value < 0.001) of the initial viability. The strongest decrease in relative viability was observed after 3× ECT treatment, supporting the notion that repetitive ECT is a very effective therapy option for CM. The median ATP concentration of the CM cells after 3× ECT treatment was 11% (SD: 11%, *p*−Value < 0.001). In detail, tumor spheroids of CRMM1 and CRMM2 cells showed only 3% (SD: 4%, *p*−Value < 10^−14^) and 8% (SD: 8%, *p*−Value < 10^−15^) of the initial viability, respectively, and that of CM2005.1 cells was 22% (SD: 12%, *p*−Value < 10^−22^) (Figure 3).

### 3.3. Repetitive ECT Treatment of CM Spheroids Is Associated with Decreased Proliferation Capacity and an Increase of Apoptotic Cells

To verify the strong cytotoxic effect as a benefit of repetitive ECT, immunocytochemical staining for Ki67 (marker of proliferating cells) and cParp-1 (marker of apoptotic cells) was performed on cross sections of tumor spheroids after 7 days of treatment.

**Figure 4:**
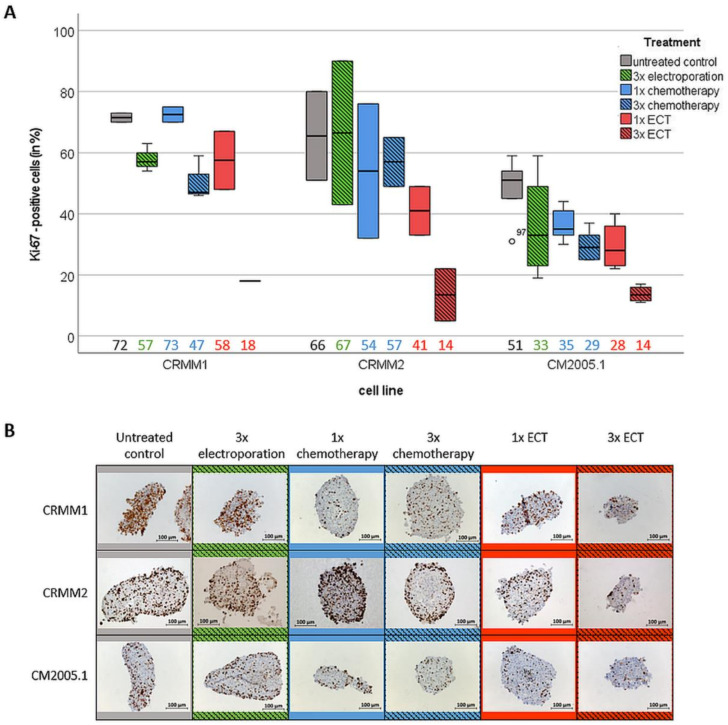
Quantitative analysis of proliferative cells using Ki67 staining in tumor spheroids on day 7 after different treatment options. (**A**) Boxplot of Ki-67 positive stained cells in cross sections of tumour spheroids after different treatment options compared to total cell count. Median values are listed on the bottom side of the boxplot; (**B**) Representative images of immunocytochemically stained spheroids after different treatment options. Scale bar = 100 µm. Abbreviation: ECT = electrochemotherapy.

Ki67-positive cells were detected in all treatment groups. However, similar to the results of spheroid size and spheroid viability, the greatest reduction of Ki67-positive cells was detected in the 3× ECT group for the three CM cell types (Ki67-positive cells < 20%) (Figure 4).

**Figure 5:**
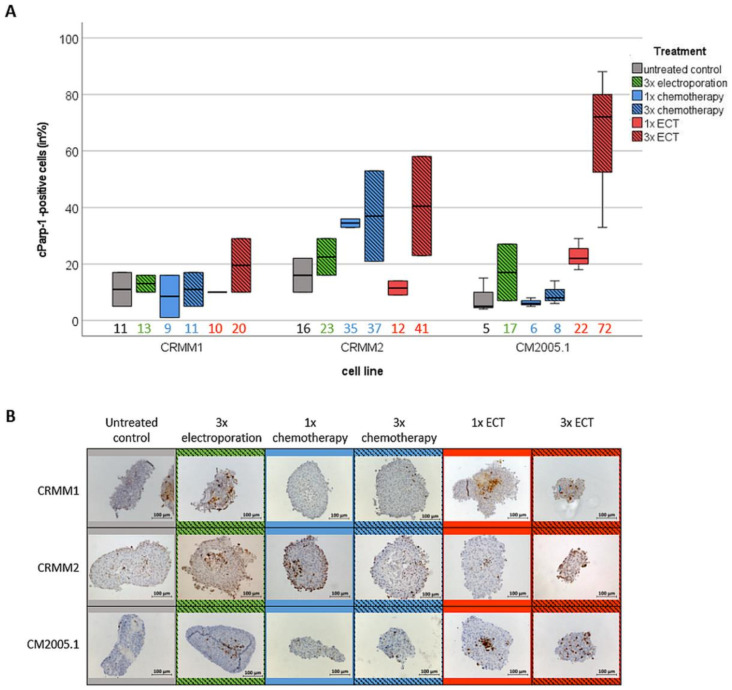
Quantitative analyses of apoptotic cells using cParp-1 staining in cross sections of tumor spheroids on day 7 after different treatment options. (**A**) Boxplot of cParp-1 positive cells in cross sections of tumor spheroids after different treatment options. Median values are listed on the bottom side of the boxplot; (**B**) Representative images of immunocytochemically stained spheroids after different treatment options. Scale bar = 100 µm. Abbreviation: ECT= electrochemotherapy.

In addition, the relative number of cParp-1-positive cells was significantly higher in 3× ECT spheroids of CM cells compared to all other treatment options. CM2005.1 cells showed the strongest increase of apoptotic cells, with 72% of cParp-1-positive cells in× ECT spheroids compared to 5% cParp-1-positive cells in untreated controls. In CRMM1, CRMM2, and CM2005.1 cells, cParp-1-positive cells increased from 11% and 16% in the untreated control group to 20% and 41% in the 3× ECT group, respectively (Figure 5).

### 3.4. Repetitive ECT-Treated Spheroids Have a Worse Outgrowth Capacity

Based on the results that viability, tumor growth and proliferation are strongly inhibited and apoptosis markers are increased in 3× ECT-treated spheroids after 7 days of treatment, we determined whether tumor spheroids are able to adhere and grow, reflecting the capacity of reproductive survival of the cells.

**Figure 6:**
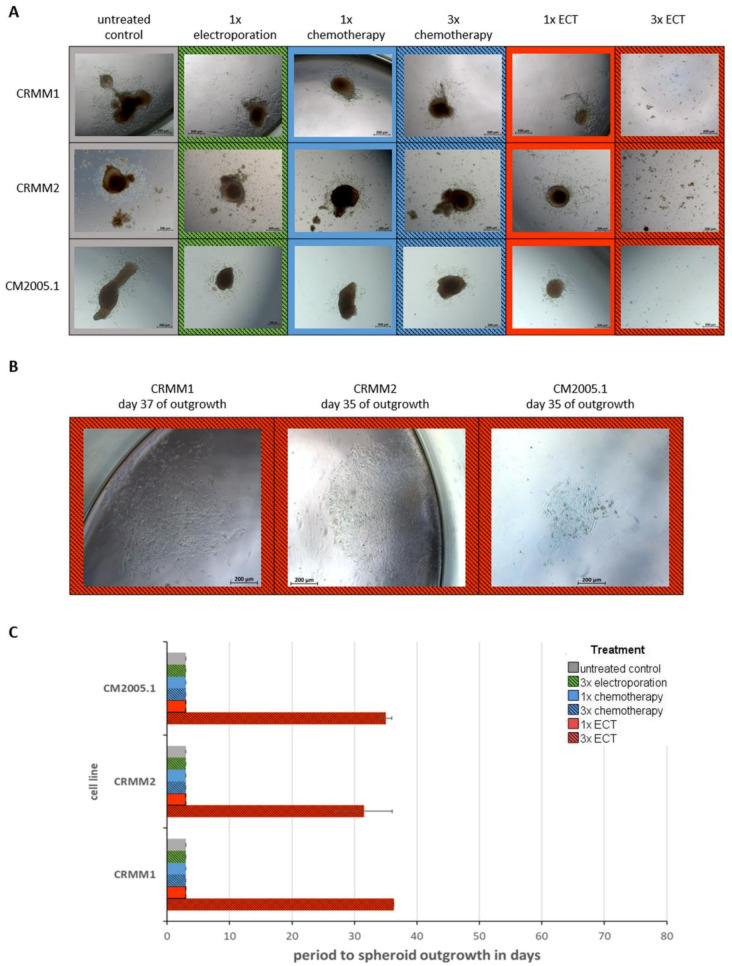
Comparison of the outgrowth capacity of 3D spheroids from the conjunctival melanoma cell lines CRMM1, CRMM2, CM2005.1 after different treatments by recultivation in cell culture plates. (**A**) Representative microscopic images of 3D spheroids on day 3 of outgrowth; (**B**) Representative microscopic images of proliferating cell colonies from 3× ECT-treated spheroids after recultivation in cell culture plates; (**C**) Comparison of the median time to outgrowth of spheroids from conjunctival cells treated with different applications. Scale bar = 200 µm. Abbreviations: ECT= electrochemotherapy.

Therefore, spheroids were carefully transferred to culture plates to allow spheroid tumor cells with the ability to adhere, migrate and proliferate the capacity to grow out of the spheroid. As a result, 100% of untreated control spheroids and the corresponding spheroids in the 3× EP, 1x, and 3× chemotherapy and 1× ECT treatment arms of each CM cell line grew out within a period of 3 days (Figure 6A). In contrast, proliferating cells could be detected in 0% of the 3× ECT treatment arm on day 3. Only in some cell culture plates did a few individual cells detach on the well bottom without any indications of proliferation in this time period. Interestingly, even prolonged cultivation of spheroids with 3× ECT application revealed a significantly reduced reproduction rate compared to the control spheroids. The first cell colonies were detectable only after a median incubation time of 35 days for CRMM1 spheroids, 32 days for CRMM2 spheroids, and 36 days for CM2005.1 spheroids (Figure 6B,C). However, only 38% of CRMM1 spheroids, 44% of CRMM2 spheroids and 33% of CM2005.1 spheroids showed such reproducing capacity within 80 days of culturing.

## 4. Discussion

This study investigated the potential improved anti-tumoral effect of ECT using repeated application using in vitro 3D tumor models of different CM cell lines. Thus, we were able to show that tumor spheroid size and viability were dramatically reduced after three ECT repetitions. Furthermore, the outgrowing capacity of repetitive ECT-treated spheroids was extremely inhibited, thus showing a significant reduction of recurrence rate using this therapy.

ECT is already an alternative antitumor therapy in the clinical management of different cancers that enhances the effectiveness of chemotherapeutic agents. The first published clinical studies of ECT involved head and neck nodules, subcutaneous and cutaneous lesions and metastases from several tumors. Currently, ECT is also applied to treat mucosal, larger, and deep-seated tumors and in internal organs. The majority of studies have been conducted using a single application of ECT with an overall comparable high objective response rate of ~80% and reduced systemic toxicity [23]. However, it has been shown that the complete response rate after single ECT treatment is ~74%, regardless of tumor histology, drug used, and route of administration, indicating that viable tumor cells can still remain [40].

Focusing on CM, only a few in vitro studies on 2D and 3D culture systems have previously reported that single ECT treatment effectively enhances chemotherapeutic effects [27,28,41]. In our study using 3D tumor spheroids from different CM cell lines, we confirmed that ECT treatment negatively affects tumor growth and viability. Further, 3D tumor cultures were significantly reduced in size and showed a formation of more loosely aggregated tumor cells with an increased amount of cell debris in the surrounding area compared to untreated controls. Nevertheless, the present study confirmed that after one ECT application, a median of 53% of tumor cells are still viable, with only minor differences in the number of proliferating cells and apoptotic cells compared to untreated controls as well as separate application of EP or chemotherapy alone. Furthermore, immediately after transferring 1x ECT-treated tumor spheroids to cell culture plates, single cells grew out quickly within three days in 100% of spheroids, comparable to untreated controls and cells treated with EP or chemotherapy alone. Translated into clinical practice, these results reflect the reality of a high risk of tumor recurrence.

Three repeated ECT (3× ECT) applications effectively increased the cytotoxic effect on 3D tumor spheroids of CM cells by inducing apoptosis, inhibiting proliferation and consequently decreasing the percentage of surviving tumor cells, as histologically and functionally confirmed. Furthermore, we showed that 3× ECT strongly reduced the outgrowth capacity of the remaining viable tumor cells and thereby decreased the risk of tumor relapse. Even if the treatment response varied slightly using different tumor cell lines, probably due to cell line-specific characteristics, the benefit of 3× ECT was dramatic. The outgrowth capacity of 3× ECT-treated spheroids supported the findings that repetitive treatment with ECT is an effective cytotoxic procedure for treating CM cells with a reduced risk of tumor recurrence or spreading. Therefore, the reculturing period of treated spheroids was prolonged up to 80 days. In the majority of 3× ECT-treated spheroids, no viable or proliferating cells were detected within 80 days of recultivation. Only 33–44% of 3× ECT-treated spheroids exhibited single-cell outgrowth and did so with a significant delay of time up to 38 days. Taken together, multiple ECT treatment sessions could create better antitumoral effectiveness. These results have been confirmed in in vivo studies on cutaneous cancers and basal cell carcinoma, which showed an improved tumor response after multiple ECT applications [20,42].

A limitation of this study is the short period of observation based on short lifespan time of the spheroids. Longer cultivation times would lead to central necrosis, affect size and viability of the spheroids. In addition, it has to be noticed that homospheroid models only consider the anti-tumor effect of ECT on tumor cells. Further studies using heterospheroids including cancer associated fibroblasts and other elements are planned to investigate the influence of tumor microenvironment on ECT sensitivity.

Due to the genetic heterogeneity within and between cancer cell lines, single tumor cells can differ in molecular characteristics. Thus, it is important to analyze which subtypes of cells may have a selective advantage and can survive multiple ECT applications. Therefore, we collected the cells that grew out from spheroids of the different treatment arms for further studies on their functional and molecular features to identify subtypes of conjunctival tumor cells with potential ECT resistance. Recent studies have supported the hypothesis that repetitive ECT treatment of tumor masses could be useful to avoid regrowth of recurrent or remaining tumor masses for better tumor control. Thus, studies on invasive ductal carcinoma, skin carcinoma, and Kaposi sarcoma have revealed long-term stable disease or complete response after multiple ECT cycles [43,44,45,46]. Importantly, Heller et al., investigated the local side effects of repeated ECT treatment in normal tissue and observed no increased toxicity or loss of function in normal skin tissue, underpinning the theory of ECT as an effective antitumor therapy [47]. Further theoretical knowledge and practical in vivo expertise are mandatory for the proper use of repeated ECT in clinical practice for managing CM.

In addition to the promising antitumor effectiveness of repeated ECT application, recent publications have reported a low side-effect profile as another advantage of this treatment modality. Electroporation causes local increased drug delivery with no further systemic effects and consequently no systemic potentiation of the cytotoxicity of the chemotherapeutic agent used [46]. Systemic or local drug delivery can increase the local concentration of the chemotherapeutic agent and therefore the local cytotoxic effect [48]. In addition, using the example of cisplatin, it has been shown that repeated ECT application did not result in acquired drug resistance in the treated area even after months of treatment time [49]. ECT is a simple cost-effective procedure with no need for extra technical skills, making it also suitable in developing countries and small hospitals, where other standard treatments are not available [23].

Overall, the data support the hypothesis that repetitive ECT results in improved antitumor effectiveness and could be a reliable alternative therapeutic option for CM. However, efficacy and kind of application depend on the type of electrodes, number of electrodes, electric field distribution, electric pulse frequency, concentration and application of the cytotoxic agent (intratumoral or systemic application) as well as biological characteristics of the treated tissue. Thus, further studies are urgently needed to establish a CM-adapted ECT protocol for good tumor control but also fewer possible side effects in adjacent ocular tissue.

## Data Availability

Not applicable.

## Supplementary Materials

The following supporting information can be downloaded at: https://www.mdpi.com/article/10.3390/jcm12031087/s1, Figure S1: Representative images of 3D spheroids of conjunctival melanoma cell line CRMM1 during treatment with electrochemotherapy.; Figure S2: Representative images of 3D spheroids of conjunctival melanoma cell line CRMM2 during treatment with electrochemotherapy.; Figure S3: Representative images of 3D spheroids of conjunctival melanoma cell line CM2005.1 during treatment with electrochemotherapy.
