# Supplementary material for: Repetitive Bleomycin-Based Electrochemotherapy Improves Antitumor Effectiveness in 3D Tumor Models of Conjunctival Melanoma
Source: J Clin Med. 2023 Jan 30;12(3):1087. doi: 10.3390/jcm12031087 (PMC9917688; doi:10.3390/jcm12031087)
Supplement: Supplementary file 1 [file jcm-12-01087-s001.zip › jcm-2096287-supplementary.pdf]

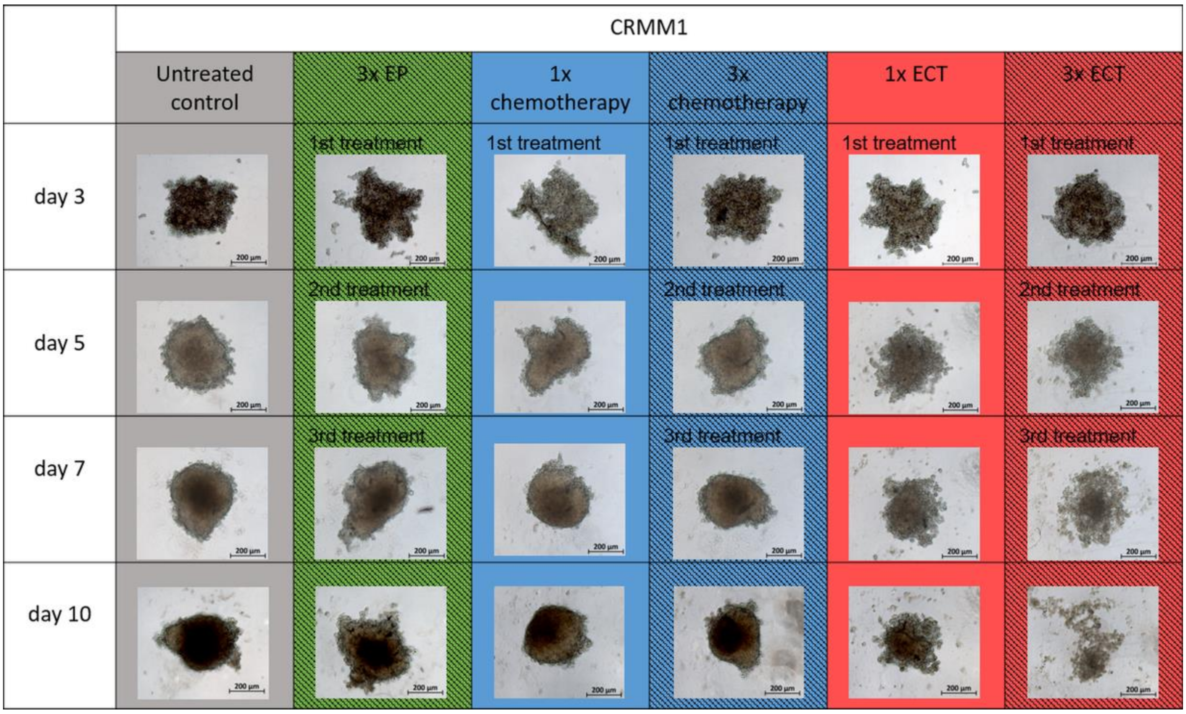

Figure S1: Representative images of 3D spheroids of conjunctival melanoma cell line CRMM1 during treatment with electrochemotherapy.

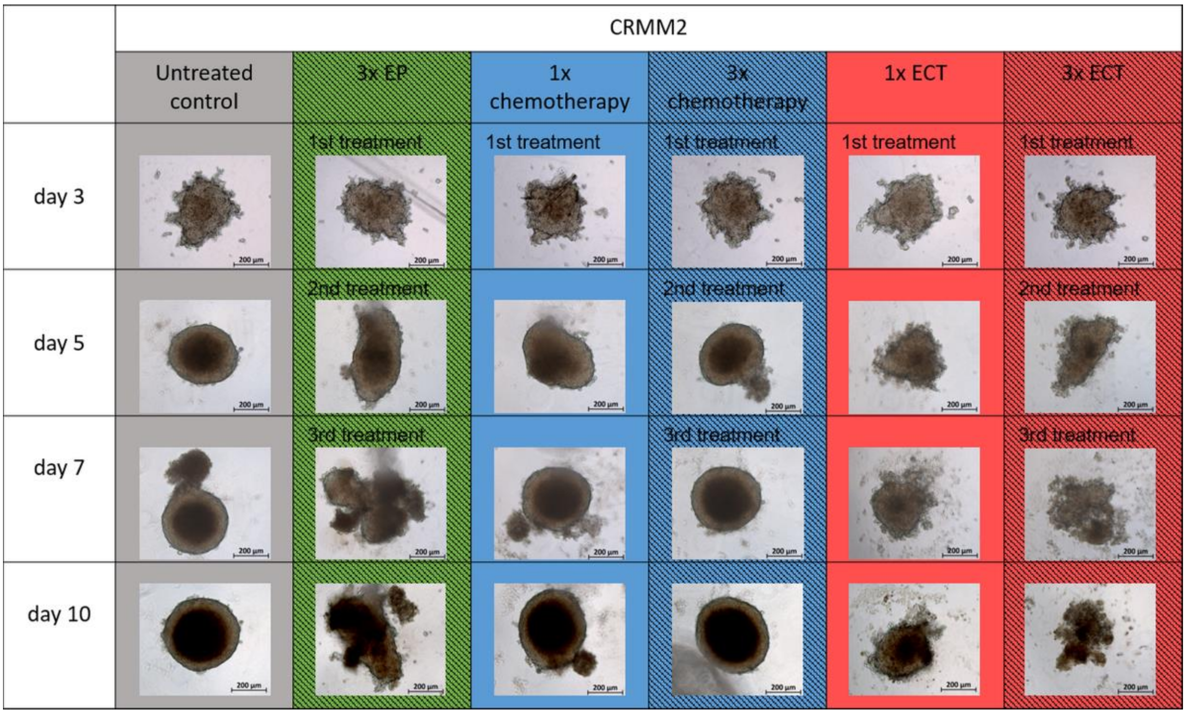

Figure S2: Representative images of 3D spheroids of conjunctival melanoma cell line CRMM2 during treatment with electrochemotherapy.

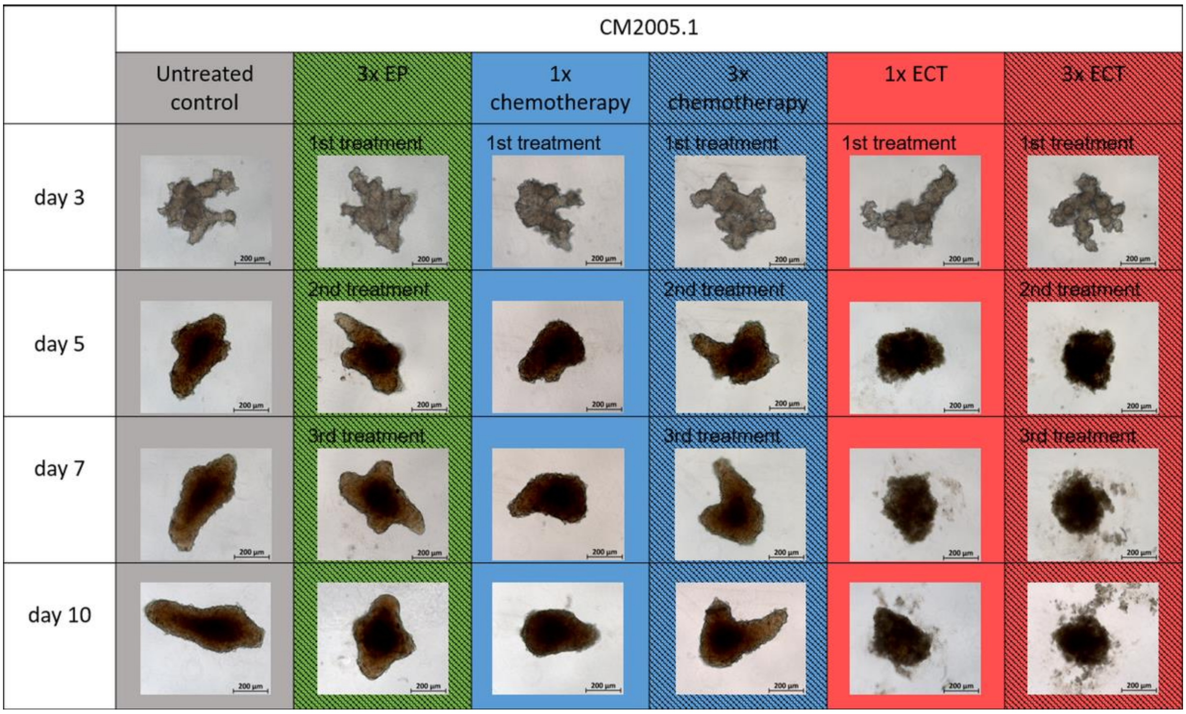

Figure S3: Representative images of 3D spheroids of conjunctival melanoma cell line CM2005.1 during treatment with electrochemotherapy.
